# (2*E*)-2-(5-Bromo-2-hy­droxy-3-meth­oxy­benzyl­idene)-*N*-phenyl­hydrazine­carbo­thio­amide

**DOI:** 10.1107/S1600536812022520

**Published:** 2012-05-23

**Authors:** Jinsa Mary Jacob, M. Sithambaresan, M. R. Prathapachandra Kurup

**Affiliations:** a Department of Applied Chemistry, Cochin University of Science and Technology, Kochi 682 022, India; b Department of Chemistry, Faculty of Science, Eastern University, Chenkalady, Sri Lanka

## Abstract

The title compound, C_15_H_14_BrN_3_O_2_S, adopts an *E*,*E* conformation with respect to the azomethine and hydrazinic bonds and exists in the thio­amide form. The two rings in the mol­ecule are twisted away from each other, making a dihedral angle of 69.13 (13)°. In the crystal, mol­ecules are linked through pairs of N—H⋯O and O—H⋯S hydrogen bonds, leading to the formation of inversion dimers which are stacked along the *a* axis. Intra­molecular N—H⋯N, O—H⋯O and C—H⋯π inter­actions are also present.

## Related literature

For applications of hydrazinecarbothio­amide and its derivatives, see: Barber *et al.* (1992 ▶); Parrilha *et al.* (2011 ▶). For the synthesis, see: Joseph *et al.* (2006 ▶). For related structures, see: Dutta *et al.* (1997 ▶); Seena *et al.* (2006 ▶, 2008 ▶); Nisha *et al.* (2011 ▶); Jacob & Kurup (2012 ▶). For C=S and C=N double-bond lengths, see: Allen *et al.* (1987) ▶.
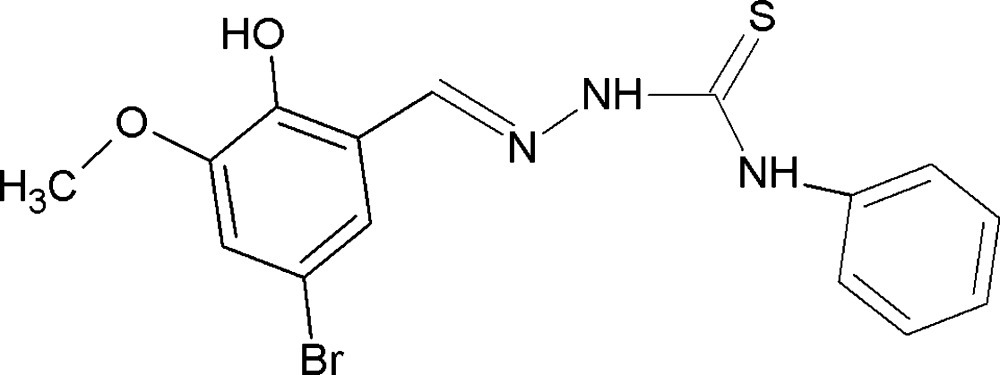


## Experimental

### 

#### Crystal data

C_15_H_14_BrN_3_O_2_S
*M* *_r_* = 380.26
Triclinic, 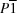

*a* = 6.1046 (5) Å
*b* = 11.0329 (8) Å
*c* = 12.4303 (9) Å
α = 101.175 (3)°
β = 91.323 (2)°
γ = 104.759 (2)°
*V* = 791.91 (10) Å^3^
*Z* = 2
Mo *K*α radiation
μ = 2.74 mm^−1^
*T* = 296 K
0.35 × 0.30 × 0.25 mm

#### Data collection

Bruker Kappa APEXII CCD diffractometer
Absorption correction: multi-scan (*SADABS*; Bruker, 2004 ▶) *T*_min_ = 0.399, *T*_max_ = 0.504
11624 measured reflections
2774 independent reflections
2338 reflections with *I* > 2σ(*I*)
*R*_int_ = 0.039

#### Refinement

*R*[*F* ^2^ > 2σ(*F* ^2^)] = 0.029
*wR*(*F* ^2^) = 0.068
*S* = 1.01
2774 reflections
212 parameters
3 restraints
H atoms treated by a mixture of independent and constrained refinement
Δρ_max_ = 0.40 e Å^−3^
Δρ_min_ = −0.44 e Å^−3^

### 

Data collection: *APEX2* (Bruker, 2004 ▶); cell refinement: *APEX2* and *SAINT* (Bruker, 2004 ▶); data reduction: *SAINT* and *XPREP* (Bruker, 2004 ▶); program(s) used to solve structure: *SIR92* (Altomare *et al.*, 1993 ▶); program(s) used to refine structure: *SHELXL97* (Sheldrick, 2008 ▶); molecular graphics: *SHELXTL* (Sheldrick, 2008 ▶) and *ORTEP-3* (Farrugia, 1997 ▶); software used to prepare material for publication: *SHELXL97* and *publCIF* (Westrip, 2010 ▶).

## Supplementary Material

Crystal structure: contains datablock(s) I, global. DOI: 10.1107/S1600536812022520/fj2553sup1.cif

Structure factors: contains datablock(s) I. DOI: 10.1107/S1600536812022520/fj2553Isup2.hkl

Supplementary material file. DOI: 10.1107/S1600536812022520/fj2553Isup3.cml

Additional supplementary materials:  crystallographic information; 3D view; checkCIF report

## Figures and Tables

**Table 1:** Hydrogen-bond geometry (Å, °) *Cg*2 is the centroid of the C9–C14 ring.

*D*—H⋯*A*	*D*—H	H⋯*A*	*D*⋯*A*	*D*—H⋯*A*
N3—H3′⋯N1	0.83 (2)	2.25 (3)	2.654 (3)	110 (2)
N2—H2⋯O2^i^	0.84 (2)	2.23 (2)	2.983 (3)	149 (2)
O2—H2′⋯O1	0.82 (2)	2.20 (3)	2.631 (2)	113 (3)
O2—H2′⋯S1^i^	0.82 (2)	2.44 (2)	3.1547 (18)	146 (3)
C15—H15*A*⋯*Cg*2^ii^	0.96	2.90	3.649 (3)	135

Symmetry codes: (i) 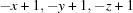; (ii) 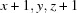.

**Fig. 1.**
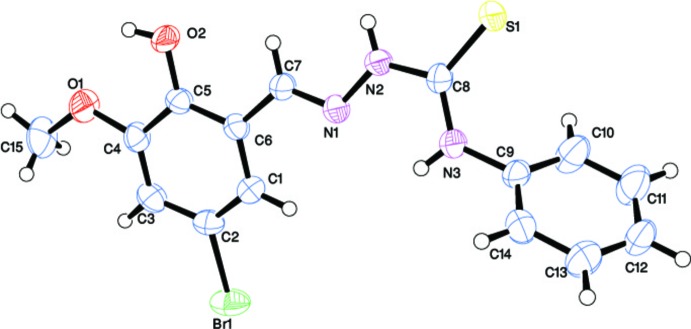
The title compound, showing 50% probability displacement ellipsoids and the atom-numbering scheme.

**Fig. 2.**
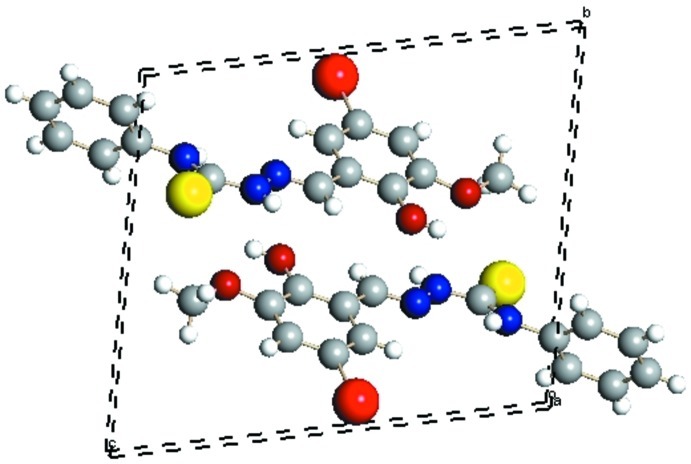
A view of the unit cell along a axis.

## supplementary crystallographic information

### Comment

The hydrazinecarbothioamides are envisaged as an important class of nitrogen-sulfur donor ligands because of their diverse chemical, biological and medicinal properties (Parrilha *et al.*, 2011). The pharmacological activity of hydrazinecarbothioamides of *o*-hydroxyaromatic aldehydes is correlated to their ability to form chelates with biologically important metal ions by bonding through O, N and S atoms (Dutta *et al.*, 1997).

The title compound adopts an *E* configuration with respect to the azomethine bond [N2—N1—C7—C6 = 174.8 (2)°] (Nisha *et al.*, 2011). Also *E* configuration is perceived about C8—N2 bond (Fig. 1) similar to 5-bromo-3-methoxysalicylaldehyde-*N*(4)-cyclohexylthiosemicarbazone (Jacob & Kurup, 2012) but in contrast to 2-hydroxyacetophenone-*N*(4)-phenylthiosemicarbazone (Seena *et al.*, 2006), where a *Z* configuration exists. This is confirmed by the N1—N2—C8—S1 torsion angle of 176.19 (18)°. Atom O1 lies *cis* to O2, with an O1—C4—C5—O2 torsion angle of -1.6 (3)° and atom N1 lies *cis* to N3, with an N1—N2—C8—N3 torsion angle of -5.9 (4)°. This favours the intramolecular hydrogen bonding interactions O2—H2'···O1 and N3—H3'···N1.

The C8—S1 bond distance [1.682 (2) Å] is closer to that expected for C═S bond length [1.60 Å] (Allen *et al.*, 1987) which confirms the existence of the compound in the thioamido form in solid state. Also the C7—N1 bond distance [1.270 (3) Å] is appreciably close to that of a C═N double bond [1.28 Å] (Allen *et al.*, 1987), confirming the azomethine bond formation.

The mean plane deviation calculations show that the molecule as a whole is non-planar. But the central hydrazinecarbothioamide group (C7/N1/N2/C8/S1/N3) is almost planar with a maximum deviation from the mean plane of 0.035 (2) Å for atom N2. This is similar to that observed in salicylaldehyde-*N*(4)-phenyl thiosemicarbazone (Seena *et al.*, 2008). The planarity of hydrazinecarbothioamide moiety allows delocalization of the π electrons throughout the C7/N1/N2/C8/S1/N3 group. The ring *Cg*1^iii^ (comprising of atoms C1—C6, with a maximum deviation of -0.011 (2) Å for C2) makes a dihedral angle of 14.80 (10)° with the hydrazinecarbothioamide moiety while the two rings in the molecule are twisted away from each other by a dihedral angle of 69.13 (13)° [symmetry code:(iii) 2 - *x*, 1 - *y*, 1 - *z*].

Fig. 2 shows the packing diagram of the title compound. The crystal packing involves two types of intramolecular hydrogen bonding interactions (Table 1), O2—H2'···O1 and N3—H3'···N1 leading to the formation of five membered rings comprising of atoms C4, C5, O2, H2' and O1 and N2, C8, N3, H3' and N1 respectively. The intermolecular hydrogen bonds N2—H2···O2^i^ and O2—H2'···S1^i^ cause the pairing of molecules leading to the formation of centrosymmetric dimers in the crystal lattice. These dimers are stacked along the *a* axis. Further stabilization is provided by non-classical C7—H7···O2 and C15—H15A···*Cg*2^ii^ interactions.

### Experimental

The title compound was prepared by adapting a reported procedure (Joseph *et al.*, 2006). To a methanolic (20 ml) solution of 4-phenylthiosemicarbazide (1 mmol, 0.1672 g), a methanolic (15 ml) solution of 5-bromo-3-methoxysalicylaldehyde (1 mmol, 0.2310 g) was added. The mixture was refluxed for 2 h in acid medium. After cooling, the compound formed was filtered off, washed with methanol and dried *in vacuo*. Yellow block shaped crystals suitable for single-crystal X-ray diffraction analysis were obtained by slow evaporation of its solution in 1:1 mixture of DMF and methanol over 3 days.

### Refinement

All H atoms on C were placed in calculated positions, guided by difference maps, with C—H bond distances 0.93–0.96 Å. H atoms were assigned as *U*_iso_=1.2Ueq (1.5 for Me). N2—H2, N3—H3' and O2—H2' H atoms were located from difference maps and restrained using *DFIX* instructions.

### Crystal data

**Table:**

C_15_H_14_BrN_3_O_2_S	*Z* = 2
*M**_r_* = 380.26	*F*(000) = 384.0
Triclinic, *P*1	*D*_x_ = 1.595 Mg m^−^^3^
Hall symbol: -P 1	Mo *K*α radiation, λ = 0.71073 Å
*a* = 6.1046 (5) Å	Cell parameters from 4882 reflections
*b* = 11.0329 (8) Å	θ = 2.8–25.9°
*c* = 12.4303 (9) Å	µ = 2.74 mm^−^^1^
α = 101.175 (3)°	*T* = 296 K
β = 91.323 (2)°	Block, yellow
γ = 104.759 (2)°	0.35 × 0.30 × 0.25 mm
*V* = 791.91 (10) Å^3^	

### Data collection

**Table:**

Bruker Kappa APEXII CCD diffractometer	2774 independent reflections
Radiation source: fine-focus sealed tube	2338 reflections with *I* > 2σ(*I*)
Graphite monochromator	*R*_int_ = 0.039
Detector resolution: 8.33 pixels mm^-1^	θ_max_ = 25.0°, θ_min_ = 2.8°
ω and φ scan	*h* = −7→7
Absorption correction: multi-scan (*SADABS*; Bruker, 2004)	*k* = −13→13
*T*_min_ = 0.399, *T*_max_ = 0.504	*l* = −14→14
11624 measured reflections	

### Refinement

**Table:**

Refinement on *F*^2^	Primary atom site location: structure-invariant direct methods
Least-squares matrix: full	Secondary atom site location: difference Fourier map
*R*[*F*^2^ > 2σ(*F*^2^)] = 0.029	Hydrogen site location: inferred from neighbouring sites
*wR*(*F*^2^) = 0.068	H atoms treated by a mixture of independent and constrained refinement
*S* = 1.01	*w* = 1/[σ^2^(*F*_o_^2^) + (0.0256*P*)^2^ + 0.4209*P*] where *P* = (*F*_o_^2^ + 2*F*_c_^2^)/3
2774 reflections	(Δ/σ)_max_ = 0.001
212 parameters	Δρ_max_ = 0.40 e Å^−^^3^
3 restraints	Δρ_min_ = −0.44 e Å^−^^3^

### Special details

**Table:**

Geometry. All e.s.d.'s (except the e.s.d. in the dihedral angle between two l.s. planes) are estimated using the full covariance matrix. The cell e.s.d.'s are taken into account individually in the estimation of e.s.d.'s in distances, angles and torsion angles; correlations between e.s.d.'s in cell parameters are only used when they are defined by crystal symmetry. An approximate (isotropic) treatment of cell e.s.d.'s is used for estimating e.s.d.'s involving l.s. planes.
Refinement. Refinement of *F*^2^ against ALL reflections. The weighted *R*-factor *wR* and goodness of fit *S* are based on *F*^2^, conventional *R*-factors *R* are based on *F*, with *F* set to zero for negative *F*^2^. The threshold expression of *F*^2^ > σ(*F*^2^) is used only for calculating *R*-factors(gt) *etc*. and is not relevant to the choice of reflections for refinement. *R*-factors based on *F*^2^ are statistically about twice as large as those based on *F*, and *R*- factors based on ALL data will be even larger.

### Fractional atomic coordinates and isotropic or equivalent isotropic displacement parameters (Å^2^)

**Table:**

	*x*	*y*	*z*	*U*_iso_*/*U*_eq_	
Br1	1.28630 (5)	0.08118 (3)	0.45166 (3)	0.05852 (12)	
S1	0.16123 (11)	0.32832 (7)	0.12714 (5)	0.05073 (19)	
O1	1.1158 (3)	0.42448 (18)	0.77355 (13)	0.0518 (5)	
O2	0.8043 (3)	0.47160 (18)	0.65009 (14)	0.0457 (4)	
N1	0.6514 (3)	0.31281 (18)	0.32779 (15)	0.0366 (5)	
N2	0.4670 (4)	0.3403 (2)	0.28201 (16)	0.0416 (5)	
N3	0.5318 (4)	0.2386 (2)	0.11374 (17)	0.0454 (5)	
C1	0.9885 (4)	0.2399 (2)	0.44657 (19)	0.0373 (5)	
H1	0.9594	0.2004	0.3726	0.045*	
C2	1.1446 (4)	0.2114 (2)	0.5112 (2)	0.0391 (6)	
C3	1.1985 (4)	0.2702 (2)	0.6213 (2)	0.0421 (6)	
H3	1.3079	0.2503	0.6629	0.051*	
C4	1.0856 (4)	0.3588 (2)	0.66732 (19)	0.0380 (5)	
C5	0.9203 (4)	0.3875 (2)	0.60356 (19)	0.0347 (5)	
C6	0.8726 (4)	0.3296 (2)	0.49340 (18)	0.0330 (5)	
C7	0.6955 (4)	0.3582 (2)	0.43026 (18)	0.0364 (5)	
H7	0.6119	0.4114	0.4660	0.044*	
C8	0.3980 (4)	0.2983 (2)	0.17484 (19)	0.0372 (5)	
C9	0.4879 (4)	0.1782 (2)	0.00017 (19)	0.0389 (6)	
C10	0.2884 (5)	0.0890 (3)	−0.0380 (2)	0.0575 (8)	
H10	0.1770	0.0683	0.0101	0.069*	
C11	0.2513 (5)	0.0295 (3)	−0.1473 (2)	0.0637 (8)	
H11	0.1133	−0.0299	−0.1728	0.076*	
C12	0.4137 (5)	0.0565 (3)	−0.2185 (2)	0.0577 (8)	
H12	0.3883	0.0155	−0.2922	0.069*	
C13	0.6148 (6)	0.1446 (4)	−0.1802 (2)	0.0722 (10)	
H13	0.7274	0.1633	−0.2282	0.087*	
C14	0.6525 (5)	0.2064 (3)	−0.0708 (2)	0.0616 (8)	
H14	0.7893	0.2669	−0.0456	0.074*	
C15	1.2722 (5)	0.3963 (3)	0.8467 (2)	0.0618 (8)	
H15A	1.2241	0.3073	0.8500	0.093*	
H15B	1.2761	0.4477	0.9188	0.093*	
H15C	1.4211	0.4150	0.8203	0.093*	
H3'	0.653 (3)	0.240 (3)	0.146 (2)	0.055 (8)*	
H2	0.389 (4)	0.380 (2)	0.3232 (19)	0.046 (8)*	
H2'	0.856 (5)	0.507 (3)	0.7130 (17)	0.072 (10)*	

### Atomic displacement parameters (Å^2^)

**Table:**

	*U*^11^	*U*^22^	*U*^33^	*U*^12^	*U*^13^	*U*^23^
Br1	0.0572 (2)	0.05035 (17)	0.0776 (2)	0.03025 (13)	0.01961 (15)	0.01303 (14)
S1	0.0438 (4)	0.0760 (5)	0.0333 (3)	0.0295 (3)	−0.0088 (3)	−0.0032 (3)
O1	0.0632 (12)	0.0653 (12)	0.0328 (9)	0.0318 (9)	−0.0119 (8)	0.0058 (9)
O2	0.0525 (11)	0.0583 (11)	0.0301 (9)	0.0324 (9)	−0.0068 (8)	−0.0039 (8)
N1	0.0374 (11)	0.0452 (11)	0.0296 (11)	0.0165 (9)	−0.0017 (8)	0.0066 (9)
N2	0.0406 (12)	0.0588 (13)	0.0292 (11)	0.0268 (10)	−0.0020 (9)	0.0006 (10)
N3	0.0448 (13)	0.0649 (14)	0.0293 (11)	0.0278 (11)	−0.0056 (10)	0.0002 (10)
C1	0.0396 (14)	0.0383 (13)	0.0334 (13)	0.0114 (10)	0.0044 (11)	0.0045 (11)
C2	0.0381 (13)	0.0375 (13)	0.0467 (15)	0.0172 (10)	0.0074 (11)	0.0104 (11)
C3	0.0392 (14)	0.0457 (14)	0.0469 (15)	0.0167 (11)	−0.0028 (11)	0.0156 (12)
C4	0.0415 (14)	0.0429 (13)	0.0308 (13)	0.0129 (11)	−0.0038 (10)	0.0090 (11)
C5	0.0345 (13)	0.0379 (12)	0.0343 (13)	0.0145 (10)	0.0015 (10)	0.0075 (10)
C6	0.0315 (12)	0.0378 (12)	0.0308 (12)	0.0112 (10)	0.0003 (10)	0.0072 (10)
C7	0.0389 (13)	0.0415 (13)	0.0296 (12)	0.0162 (10)	−0.0015 (10)	0.0026 (10)
C8	0.0397 (14)	0.0424 (13)	0.0292 (12)	0.0135 (11)	−0.0024 (10)	0.0036 (10)
C9	0.0459 (15)	0.0467 (14)	0.0287 (12)	0.0245 (12)	−0.0014 (11)	0.0027 (11)
C10	0.0643 (19)	0.0545 (17)	0.0432 (16)	0.0037 (14)	0.0135 (14)	−0.0002 (14)
C11	0.066 (2)	0.0541 (17)	0.0534 (18)	0.0012 (15)	0.0036 (16)	−0.0103 (15)
C12	0.070 (2)	0.0685 (19)	0.0329 (14)	0.0286 (16)	−0.0015 (14)	−0.0053 (13)
C13	0.059 (2)	0.112 (3)	0.0384 (16)	0.0219 (19)	0.0125 (14)	−0.0017 (17)
C14	0.0383 (15)	0.092 (2)	0.0448 (16)	0.0143 (15)	−0.0023 (13)	−0.0035 (16)
C15	0.076 (2)	0.0678 (19)	0.0430 (16)	0.0209 (16)	−0.0209 (15)	0.0144 (14)

### Geometric parameters (Å, º)

**Table:**

Br1—C2	1.900 (2)	C4—C5	1.404 (3)
S1—C8	1.682 (2)	C5—C6	1.384 (3)
O1—C4	1.361 (3)	C6—C7	1.454 (3)
O1—C15	1.434 (3)	C7—H7	0.9300
O2—C5	1.358 (3)	C9—C10	1.363 (4)
O2—H2'	0.819 (18)	C9—C14	1.369 (4)
N1—C7	1.270 (3)	C10—C11	1.376 (4)
N1—N2	1.376 (3)	C10—H10	0.9300
N2—C8	1.342 (3)	C11—C12	1.359 (4)
N2—H2	0.843 (17)	C11—H11	0.9300
N3—C8	1.337 (3)	C12—C13	1.365 (4)
N3—C9	1.427 (3)	C12—H12	0.9300
N3—H3'	0.830 (17)	C13—C14	1.383 (4)
C1—C2	1.366 (3)	C13—H13	0.9300
C1—C6	1.404 (3)	C14—H14	0.9300
C1—H1	0.9300	C15—H15A	0.9600
C2—C3	1.389 (3)	C15—H15B	0.9600
C3—C4	1.379 (3)	C15—H15C	0.9600
C3—H3	0.9300		
			
C4—O1—C15	117.7 (2)	C6—C7—H7	119.0
C5—O2—H2'	112 (2)	N3—C8—N2	115.7 (2)
C7—N1—N2	114.91 (19)	N3—C8—S1	125.34 (18)
C8—N2—N1	122.0 (2)	N2—C8—S1	118.95 (18)
C8—N2—H2	118.8 (18)	C10—C9—C14	119.4 (2)
N1—N2—H2	119.1 (18)	C10—C9—N3	121.1 (2)
C8—N3—C9	126.3 (2)	C14—C9—N3	119.4 (2)
C8—N3—H3'	115 (2)	C9—C10—C11	120.3 (3)
C9—N3—H3'	118 (2)	C9—C10—H10	119.9
C2—C1—C6	119.1 (2)	C11—C10—H10	119.9
C2—C1—H1	120.4	C12—C11—C10	120.8 (3)
C6—C1—H1	120.4	C12—C11—H11	119.6
C1—C2—C3	122.6 (2)	C10—C11—H11	119.6
C1—C2—Br1	119.52 (18)	C11—C12—C13	119.0 (3)
C3—C2—Br1	117.78 (17)	C11—C12—H12	120.5
C4—C3—C2	118.3 (2)	C13—C12—H12	120.5
C4—C3—H3	120.8	C12—C13—C14	120.6 (3)
C2—C3—H3	120.8	C12—C13—H13	119.7
O1—C4—C3	126.1 (2)	C14—C13—H13	119.7
O1—C4—C5	113.7 (2)	C9—C14—C13	119.8 (3)
C3—C4—C5	120.2 (2)	C9—C14—H14	120.1
O2—C5—C6	119.51 (19)	C13—C14—H14	120.1
O2—C5—C4	120.0 (2)	O1—C15—H15A	109.5
C6—C5—C4	120.5 (2)	O1—C15—H15B	109.5
C5—C6—C1	119.2 (2)	H15A—C15—H15B	109.5
C5—C6—C7	119.2 (2)	O1—C15—H15C	109.5
C1—C6—C7	121.5 (2)	H15A—C15—H15C	109.5
N1—C7—C6	122.1 (2)	H15B—C15—H15C	109.5
N1—C7—H7	119.0		
			
C7—N1—N2—C8	−179.5 (2)	C2—C1—C6—C7	−176.4 (2)
C6—C1—C2—C3	−1.7 (4)	N2—N1—C7—C6	174.8 (2)
C6—C1—C2—Br1	175.39 (17)	C5—C6—C7—N1	176.6 (2)
C1—C2—C3—C4	1.4 (4)	C1—C6—C7—N1	−6.6 (4)
Br1—C2—C3—C4	−175.76 (18)	C9—N3—C8—N2	175.5 (2)
C15—O1—C4—C3	−2.7 (4)	C9—N3—C8—S1	−6.8 (4)
C15—O1—C4—C5	176.6 (2)	N1—N2—C8—N3	−5.9 (4)
C2—C3—C4—O1	179.5 (2)	N1—N2—C8—S1	176.19 (18)
C2—C3—C4—C5	0.2 (4)	C8—N3—C9—C10	−52.8 (4)
O1—C4—C5—O2	−1.6 (3)	C8—N3—C9—C14	129.9 (3)
C3—C4—C5—O2	177.8 (2)	C14—C9—C10—C11	−1.2 (4)
O1—C4—C5—C6	179.1 (2)	N3—C9—C10—C11	−178.4 (3)
C3—C4—C5—C6	−1.5 (4)	C9—C10—C11—C12	1.4 (5)
O2—C5—C6—C1	−178.2 (2)	C10—C11—C12—C13	−0.5 (5)
C4—C5—C6—C1	1.2 (4)	C11—C12—C13—C14	−0.4 (5)
O2—C5—C6—C7	−1.2 (3)	C10—C9—C14—C13	0.2 (4)
C4—C5—C6—C7	178.1 (2)	N3—C9—C14—C13	177.5 (3)
C2—C1—C6—C5	0.4 (3)	C12—C13—C14—C9	0.6 (5)

### Hydrogen-bond geometry (Å, º)

*Cg*2 is the centroid of the C9–C14 ring.

**Table:**

*D*—H···*A*	*D*—H	H···*A*	*D*···*A*	*D*—H···*A*
N3—H3′···N1	0.83 (2)	2.25 (3)	2.654 (3)	110 (2)
N2—H2···O2^i^	0.84 (2)	2.23 (2)	2.983 (3)	149 (2)
O2—H2′···O1	0.82 (2)	2.20 (3)	2.631 (2)	113 (3)
O2—H2′···S1^i^	0.82 (2)	2.44 (2)	3.1547 (18)	146 (3)
C15—H15*A*···*Cg*2^ii^	0.96	2.90	3.649 (3)	135
C7—H7···O2	0.93	2.44	2.764 (3)	101

Symmetry codes: (i) −*x*+1, −*y*+1, −*z*+1; (ii) *x*+1, *y*, *z*+1.
